# Beta-Blockers as an Immunologic and Autonomic Manipulator in Critically Ill Patients: A Review of the Recent Literature

**DOI:** 10.3390/ijms25158058

**Published:** 2024-07-24

**Authors:** Akram M. Eraky, Yashwanth Yerramalla, Adnan Khan, Yasser Mokhtar, Mostafa Alamrosy, Amr Farag, Alisha Wright, Matthew Grounds, Nicole M. Gregorich

**Affiliations:** 1 Emergency Medicine, Freeman Health System, Joplin, MO 64804, USA; drwright2b@hotmail.com (A.W.); mdgrounds@yahoo.com (M.G.); 2 Medical Education Department, Kansas City University, Kansas City, MO 64106, USA; 3 Critical Care Medicine, Freeman Health System, Joplin, MO 64804, USA; yerray02@gmail.com (Y.Y.); akhan3@freemanhealth.com (A.K.); ymmokhtar@freemanhealth.com (Y.M.); 4 Cardiology and Angiology Unit, Department of Clinical and Experimental Internal Medicine, Medical Research Institute, Alexandria University, Alexandria 5422031, Egypt; mostafa201248@gmail.com; 5 Critical Care Medicine, Portsmouth University Hospital, Portsmouth PO6 3LY, UK; dr.amrmansour1262@gmail.com; 6 School of Medicine and Public Health, University of Wisconsin, Madison, WI 53726, USA; ngregorich@wisc.edu

**Keywords:** beta-blockers, adrenergic receptors, septic shock, electric storm, cardiogenic shock

## Abstract

The autonomic nervous system plays a key role in maintaining body hemostasis through both the sympathetic and parasympathetic nervous systems. Sympathetic overstimulation as a reflex to multiple pathologies, such as septic shock, brain injury, cardiogenic shock, and cardiac arrest, could be harmful and lead to autonomic and immunologic dysfunction. The continuous stimulation of the beta receptors on immune cells has an inhibitory effect on these cells and may lead to immunologic dysfunction through enhancing the production of anti-inflammatory cytokines, such as interleukin-10 (IL-10), and inhibiting the production of pro-inflammatory factors, such as interleukin-1B IL-1B and tissue necrotizing factor-alpha (TNF-alpha). Sympathetic overstimulation-induced autonomic dysfunction may also happen due to adrenergic receptor insensitivity or downregulation. Administering anti-adrenergic medication, such as beta-blockers, is a promising treatment to compensate against the undesired effects of adrenergic surge. Despite many misconceptions about beta-blockers, beta-blockers have shown a promising effect in decreasing mortality in patients with critical illness. In this review, we summarize the recently published articles that have discussed using beta-blockers as a promising treatment to decrease mortality in critically ill patients, such as patients with septic shock, traumatic brain injury, cardiogenic shock, acute decompensated heart failure, and electrical storm. We also discuss the potential pathophysiology of beta-blockers in various types of critical illness. More clinical trials are encouraged to evaluate the safety and effectiveness of beta-blockers in improving mortality among critically ill patients.

## 1. Introduction

The autonomic nervous system (ANS) maintains unconscious control of body hemostasis in both physiologic and pathologic conditions. ANS is considered a part of the central nervous system (CNS) with central regulation by the hypothalamus and two efferent branches, including the parasympathetic (PNS) and sympathetic nervous (SNS) systems. Both PNS and SNS have preganglionic and postganglionic neurons. Additionally, both have acetylcholine-medicated nicotinic receptors in the ganglion between the preganglionic and postganglionic neurons [1,2,3,4,5]. 

Postganglionic nerves in the PNS are cholinergic and formed of several nerves, such as the pelvic splanchnic, vagus, and glossopharyngeal nerves. Moreover, their effect is achieved by activating the muscarinic receptors on the target organs. In contrast, SNS is characterized by the presence of adrenergic receptors on the target organs to mediate its sympathetic effects [3,4,5,6]. Adrenergic receptors include alpha receptors and beta receptors. From a cardiovascular standpoint, alpha-1 receptors are mainly located on the blood vessels and responsible for vasoconstriction. Presynaptic alpha-2 receptor activation inhibits norepinephrine release and has sympatholytic effects, while alpha-2 receptors located in the smooth muscle cells of peripheral arteries have the same effect as alpha-1 receptors. On the other hand, beta-1 and beta-2 receptors are mainly located on the myocardium where their activation causes positive chronotropic, dromotropic, and inotropic effects. Beta-2 receptors are also located on blood vessels and their activation causes vasodilation [5,6,7,8,9,10,11,12].

**Figure 1:**
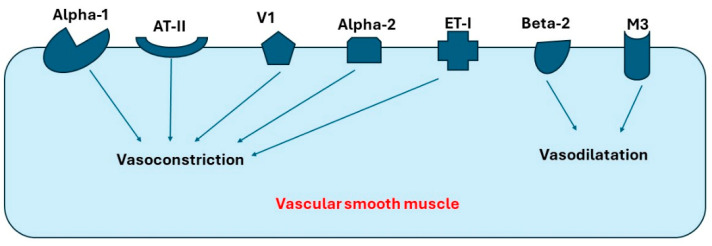
**Receptors in blood vessels that cause vasoconstriction or vasodilatation**. Abbreviations: AT-II, angiotensin-II; V1, Vasopressin-1 receptor; ET-I, Endothelin-I receptor; M3, Muscarinic-3 receptor.

Many receptors expressed in blood vessels can affect blood pressure, preload, and afterload through vasoconstriction or vasodilatation. Those receptors include alpha-1, alpha-2, beta-2, muscarinic receptor-3 (M-3), vasopressin-1a (V1a), endothelin-1 (ET1), and angiotensin-II (AT-II). Moreover, V1a and ET-1 have been found to be the most common receptors in both coronary arteries and pulmonary artery, while beta receptors have the lowest expression levels in these arteries [11,12,13,14]. Interestingly, alpha-2 receptors have higher expression in the coronary artery compared to other arteries. As a result, Liu et al. suggest that agents blocking alpha-2 receptors can present a novel therapy for ischemic cardiomyopathy [12]. In summary, receptors expressed in arterial blood vessels can be classified into the following: (1) receptors causing vasoconstriction, such as alpha-1, alpha-2, AT-II, and V-I, and (2) receptors causing vasodilation, such as M3 and beta-2. Figure 1 demonstrates the important receptors expressed in the blood vessels and their primary function.

Overstimulation of the sympathetic nervous system as a compensating mechanism in many pathologies can lead to autonomic and immunologic dysfunction. This may result in increased mortality. [15,16,17]. The idea of beta-blocker administration in patients with these pathologies to offset the adverse effects of the adrenergic surge and decrease mortality is a new trend in the literature. A balance between the benefits of compensating sympathetic stimulation and the disadvantages of sympathetic overstimulation, which could be minimized by beta-blockers, should be sought. Herein, we demonstrate the pathophysiology of autonomic and immunologic dysfunction due to high extrinsic or intrinsic catecholamine levels. Moreover, we discuss using beta-blockers as a promising treatment to decrease mortality in septic shock, electrical storm, cardiogenic shock (CS), acute decompensated heart failure (ADHF), and traumatic brain injury (TBI). 

## 2. Discussion

### 2.1. Effect of Beta-Blockers in Patients with Septic Shock 

Sepsis is defined as life-threatening organ dysfunction resulting from a dysregulated reaction to an infection. Beginning with the first published guidelines in 2004, the definition of sepsis and the guidelines of its treatment have been revisited over the last two decades. The second guidelines were published in 2008 and the third guidelines appeared in 2013, while the last revised guidelines were published in 2021 [18,19,20,21,22]. This highlights the importance of and the need for the continuous revision of sepsis treatment. 

In septic shock, ANS responds to restore hemostasis by activating SNS and increasing the level of circulating catecholamines. The response of SNS can be classified into short-term reflexes that happen in seconds to minutes to maintain blood flow to the vital organs by increasing the sympathetic outflow to peripheral vessels and the heart, and long-term reflexes to maintain vascular tone and blood volume through the renin–angiotensin–aldosterone system [5,6,15,23]. 

Short-term reflexes are mediated by baroreceptors located in the aortic arch, carotid sinuses, cardiac atria and ventricles, large systemic veins, and pulmonary arteries. Short term-reflexes may also include increasing tidal volume and respiratory rate in response to hypoxia detected by peripheral chemoreceptors in the aortic arch and carotid bodies, or hypercapnia detected by central chemoreceptors in medulla [23,24,25,26,27]. Although ANS has a significant role as a compensating mechanism in septic shock and ANS dysfunction has a undesired effect in increasing mortality, recent guidelines of sepsis management do not include the role of ANS dysfunction manipulation [19].

The first-line treatment of septic shock is fluid administration to counteract hypotension. Moreover, vasopressors are fundamental therapy in septic shock to reverse sepsis-induced vasodilatation to improve organ perfusion [20,28]. Many controversies still exist about the management of sepsis, such as whether early use of norepinephrine may improve mortality and morbidity compared to late use, whether novel treatments such as angiotensin II should be used, whether using a beta-blocker is beneficial in those patients, and whether different MAP values should be targeted [20,29,30]. More research effort should be directed to discuss these controversies.

#### 2.1.1. Vasopressors and Adrenergic Stimulation as a Treatment of the Septic Shock 

The first-line vasopressor in the management of septic shock is norepinephrine which is considered the most commonly used vasopressor. It causes strong alpha 1-induced vasoconstriction and beta 1-induced chronotropy and inotropy. Vasopressin is considered the second-line vasopressor in septic shock [19,20]. Vasopressin does not work on alpha or beta receptors, but rather stimulates V receptors, resulting in increased venous and arterial tone (by activating V1a receptor) and water retention (by activating V2 receptor). However, vasopressin can induce corticotropic axis stimulation (by activating V1b receptor), insulin secretion (by activating V1b receptor), and release of coagulation factors (by activating V2 receptor) that may lead to major side effects [28,31]. Of interest, V1a is the most expressed receptor in the coronary, renal, mesenteric, and peripheral arteries [12]. 

In the VASST trial, vasopressin was found to be more effective than norepinephrine in less severe septic shock [31]. In a recent meta-analysis, there was no difference found between norepinephrine and vasopressin as a treatment of septic shock regarding adverse effects, mortality, and duration of hospitalization. Of interest, vasopressin was found to have lower chances of requiring renal replacement therapy [32]. Adding vasopressin to norepinephrine decreases norepinephrine dosage; subsequently, it decreases the consequences of excessive adrenergic load. For these reasons, adding vasopressin to epinephrine before reaching the maximum dose of epinephrine is appealing. According to 2021 guidelines, adding vasopressin is recommended in patients with septic shock on norepinephrine who did not achieve adequate MAP. This is preferred over escalating the dose of norepinephrine. Vasopressin is usually started when the norepinephrine dose ranges from 0.25 to 0.5 mg/kg/min [19,31,32]. 

Terlipressin is another vasopressor that has a stronger effect on V1 receptors compared to V2 receptors. In a large randomized clinical trial, there was no difference in mortality between terlipressin and norepinephrine. However, terlipressin was associated with more side effects [33]. According to the 2021 guidelines, terlipressin is not suggested in septic shock [19]. Another selective vasopressin agonist is selepressin which works on V1a receptors. In the SEPSIS-ACT trial, adding selepressin to norepinephrine did not show any improvement in vasopressor-free days or ventilator-free days within 30 days compared to adding placebo [34]. 

Epinephrine is derived from the Greek words, “epi”, which means on, and “nephros”, which means kidney, while adrenaline is derived from the Latin words, “ad”, which means near, and “renes”, which means kidney. It is considered another second-line vasopressor. According to the 2021 guidelines, epinephrine addition is recommended for patients who are on both norepinephrine and vasopressin but have inadequate MAP [19]. Epinephrine works on alpha-1, beta-1, and beta-2 receptors. Its beta-2 effect makes it suitable for patients who need bronchodilatation. Epinephrine has been used in the treatment of anaphylactic shock, severe croup, and cardiac arrest [35,36]. Per the 2021 guidelines, dobutamine can be added to norepinephrine to treat patients with septic shock and cardiac dysfunction who have persistent hypoperfusion despite adequate blood pressure and volume status. Of interest, epinephrine has the effect of both dobutamine and norepinephrine combined and can be used alone in patients with septic shock and cardiac dysfunction [19,28]. The concept of epinephrine having the same effect of dobutamine plus norepinephrine was backed up by a recent meta-analysis of 12 randomized clinical trials [37]. However, using epinephrine is associated with worse outcomes and increased mortality, compared to using norepinephrine and dobutamine combined. As a result, the combination of norepinephrine plus dobutamine is preferred over epinephrine alone [35].

#### 2.1.2. Catecholamine-Induced Immunologic Dysfunction in Septic Shock

Overstimulation of adrenergic receptors is associated with increased mortality and morbidity in critically ill patients [15]. Worse outcomes are found to be associated with higher levels of endogenous or exogenous catecholamine exposure [37,38,39,40]. This undesirable effect of excessive adrenergic effect could be due to catecholamine-induced immunologic and autonomic dysfunction [15,41,42]. 

Theoretically, catecholamines can have a negative effect on immune system because beta receptors exist on innate immune cells, such as neutrophils and macrophages, and adaptive immune cells, such as T-lymphocytes, B-lymphocytes, and natural killer cells. These beta receptors have an inhibitory effect on the immune cells. Continuous stimulation of these receptors may result in immunologic dysfunction [16,41,43,44,45]. In previous in vitro and in vivo studies, norepinephrine was reported to have an inhibitory effect on the production of pro-inflammatory cytokines and a stimulating effect on the production of anti-inflammatory cytokines, such as interleukin-10 (IL-10) [46,47]. 

In a recent study by Stolk et al., the effects of norepinephrine and vasopressin on leukocytes were evaluated using in vitro models, animal samples, and human samples. In their in vitro models, they used lipopolysaccharides (LPS) to stimulate leukocytes in the presence of norepinephrine or vasopressin. They found that norepinephrine stimulated the anti-inflammatory effect of immune cells by decreasing the production of proinflammatory cytokines, such as interleukin-1B (IL-1B) and tissue-necrotizing factor-alpha (TNF-alpha) and augmenting the anti-inflammatory response of IL-10. On testing both the isolated monocytes sample and the whole blood sample, they found the same results. Of interest, vasopressin was not associated with these immunologic changes. They also found that reactive oxygen species (ROS) production was inhibited in samples with norepinephrine, while vasopressin did not affect ROS production [16]. Next, the beta-blocker propranolol was added to these samples. Interestingly, propranolol successfully offset the effects of norepinephrine on the production of the inflammatory cytokines and TNF-alpha. In contrast, the alpha-1 blocker prazosin and the alpha-2 blocker yohimbine did not have a statistically significant effect on the epinephrine-induced effect on TNF-alpha. However, yohimbine reversed the effects of epinephrine on IL-10. In mice, LPS-challenged healthy humans, and patients with septic shock, results similar to the in vitro experiment were found [16]. These findings suggest that epinephrine-induced immune dysfunction is mainly modulated by stimulating beta receptors.

Stolk et al. also found that blocking beta-2 receptors has an effective and protective role against epinephrine-induced immune dysfunction, while blocking beta-1 receptors does not have a significant role in reversing epinephrine-induced immune dysfunction [16]. These findings are consistent with earlier studies showing that immune dysfunction may be induced by beta-2 receptor stimulation. In a previous study by Agac, norepinephrine was found to suppress TNF-alpha. This effect was successfully reversed by the non-selective beta-blocker nadolol. However, the non-selective alpha-blocker phentolamine could not suppress norepinephrine-induced TNF-alpha suppression. They also found that a beta-2 antagonist successfully reversed norepinephrine-induced TNF-alpha suppression, while the beta-1 antagonist atenolol could not reverse this effect [48]. This autonomic dysfunction was also noticed with other agents that stimulate beta-2 receptors, such as epinephrine, zilpaterol, and clenbuterol [47,49,50]. In contrast, catecholamines, such as dobutamine, that primarily affect beta-1 were not found to affect the innate immune system [51]. This demonstrates the potential protective role of beta-2 blockers to offset epinephrine-induced autonomic dysfunction. Catecholamine-induced immunologic dysfunction may explain why patients who require extrinsic catecholamines, such as norepinephrine and epinephrine, develop higher mortality and morbidity compared to patients not taking extrinsic catecholamines.

**Figure 2:**
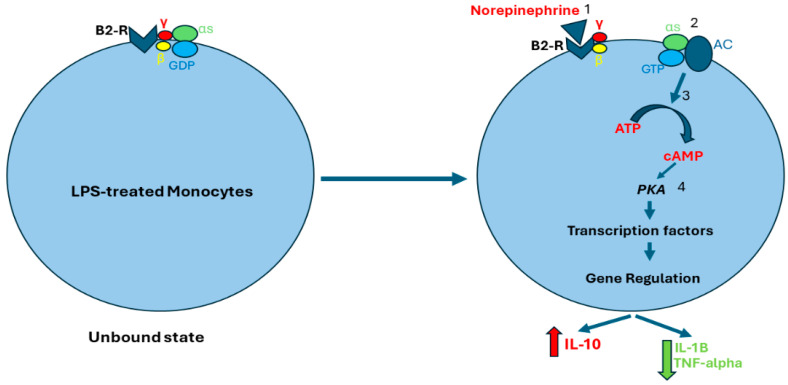
**Norepinephrine-induced activation of beta-2 receptors**. Norepinephrine binds to beta-2 receptors (1), which stimulates the exchange of GDP for GTP. As a result, Gαs and Gβγ separate (2). The GTP–Gαs complex activates AC which stimulates cAMP production (3). cAMP activates PKA which regulates gene transcription of both anti-inflammatory and pro-inflammatory cytokines (4). Abbreviations: B2-R, Beta-2 receptor; S, G protein alpha s-subunit; γ, G protein gamma subunit; β, G protein beta subunit; GDP, guanosine diphosphate; GTP, guanosine triphosphate; AC, adenyl cyclase; ATP, adenosine triphosphate; cAMP, cyclic adenosine monophosphate; PKA, protein kinase A.

Beta-2 receptors are coupled to G protein alpha s-subunit (Gαs). Norepinephrine binds to beta-2 receptors, leading to guanosine diphosphate (GDP) exchange with guanosine triphosphate (GTP), and Gαs and G protein beta/gamma subunit (Gβγ) uncoupling. The GTP–Gαs complex activates adenyl cyclase (AC) which stimulates adenosine triphosphate (ATP) conversion to cyclic adenosine monophosphate (cAMP), which then activates protein kinase A (PKA). Subsequently, PKA regulates gene transcription, resulting in enhanced production of anti-inflammatory cytokines, such as IL-10, and inhibiting the production of pro-inflammatory factors, such as IL-1B and TNF-alpha (Figure 2) [16,52,53,54]. 

#### 2.1.3. Catecholamine-Induced Autonomic Dysfunction in Septic Shock

Excessive adrenergic effects may also result in SNS dysfunction. Overstimulation of adrenergic receptors in septic shock is thought to lead to desensitization and downregulation of adrenergic receptors. Overproduction of nitric oxide and cytokine release may also lead to the same effect. Subsequently, the body will lose the compensatory role of SNS, leading to deterioration of the blood pressure despite the presence of high levels of catecholamines in the blood [15,17,55,56,57,58,59]. Theoretically, decatecholaminisation by administering beta-blockers may reverse this effect by inducing adrenergic receptor upregulation and enhancing receptor sensitivity [22,24,25,26,27,28].

The first mechanism of catecholamine-induced autonomic dysfunction is adrenergic receptor insensitivity or downregulation. In animal studies, beta and alpha receptors were found to undergo a biphasic expression during sepsis. Compared to the early stage of sepsis, increased adrenergic effect increases transcription of the Gαi-2 protein that stimulates the sympatholytic effect of alpha-2 receptors at the late stage of sepsis, while there was no change noticed between early sepsis and late sepsis phases in the expression of Gαs and Gβ proteins. This may augment the sympatholytic effect of alpha-2 receptors. Of interest, AC activity in rat hearts did not change during the early sepsis, but it did decrease during late sepsis [10,60]. The steady expression of Gαs and Gβ proteins, along with decreased AC activity in late sepsis, could be explained by developing decreased sensitivity in beta receptors after prolonged exposure to high levels of catecholamines. Interestingly, isoproterenol-stimulated AC activity does not change during early sepsis, while it decreases during the late phase of sepsis. This indicates that the effect of the alpha and beta subunits decreases during the late phase of sepsis [10,60]. In a clinical study by Bernardin et al., patients with septic shock or severe sepsis were found to have post-receptor defects of the beta receptor signal transduction [57]. This is consistent with the same findings in the aforementioned animal study. 

**Figure 3:**
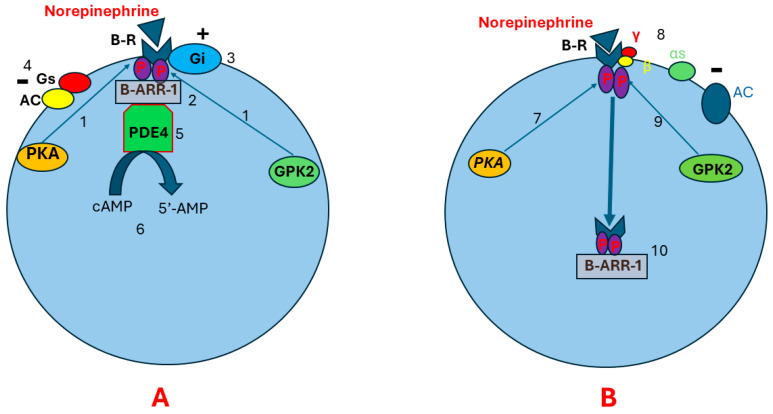
**Pathophysiology of beta receptor insensitivity due to chronic stimulation by norepinephrine**. Illustration (**A**) shows that continuous stimulation of beta receptors results in beta receptor phosphorylation in two different sites by PKA and GPK (1). This leads to conformational changes in the beta receptor that allow the scaffolding protein B-ARR-1 to bind to the beta receptor (2). As a result, more conformational changes happen in the beta receptor, leading to enhanced beta receptor coupling to Gi protein (3) and impaired Gs binding to the beta receptor (4). Subsequently, AC activity will be suppressed and cAMP production decreases. Beta receptor–B-ARR-1 complex induces cnPDE recruitment and attachment to B-ARR-1 (5). The cnPDE hydrolyzes cAMP to 5’-AMP, resulting in degrading the pre-existing cAMP (6). Illustration (**B**) shows that receptor downregulation and desensitization may also happen through receptor internalization. After phosphorylation of beta receptors by PKA (7), Gαs and Gβγ uncoupling happens (8). This allows GPK2 to bind to Gβγ (9), leading to beta receptor phosphorylation at two different sites. Receptor phosphorylation in two different sites results in conformational changes in beta receptors. These conformational changes allow B-ARR-1 to bind to the receptor (10). B-ARR-1 induces receptor internalization (10). The internalized receptor is transported to lysosomes for degradation. Abbreviations: B-R, beta receptor; AC, adenyl cyclase; αs, cAMP, cyclic adenosine monophosphate; PKA, protein kinase A; GPK2, G protein-coupled kinase-2; P, phosphorus; B-ARR-1, B-arrestin-1; cnPDE4, cyclic nucleotide phosphodiesterase 4; 5’-AMP, 5’- adenosine monophosphate; G protein alpha s-subunit; γ, G protein gamma subunit; β, G protein beta subunit.

Continuous stimulation of beta receptors by catecholamines may lead to receptor desensitization and downregulation. Receptor desensitization happens through phosphorylation of beta receptors by PKA, leading to Gαs and Gβγ uncoupling. This allows G protein-coupled kinase-2 (GPK2) to bind to Gβγ. Beta receptor phosphorylation at two different sites by GPK2 and PKA results in conformational changes in beta receptors that allow a scaffolding protein called B-arrestin-1 to bind to the beta receptors. The B-arrestin plus Beta receptor complex leads to conformational changes in beta receptors [61,62,63,64,65,66]. This may result in three consequences. First, the new conformational changes enhance beta receptor coupling to Gi protein and impair Gs binding to the beta receptors. This suppresses AC activity and prevents cAMP production [67,68]. Second, the beta receptor plus B-arrestin complex recruits cyclic nucleotide phosphodiesterase (cnPDE) to attach to this complex, leading to the hydrolyzation of cAMP to 5’-adenosine monophosphate (5’-AMP). This results in degradation of the pre-existing cAMP [69,70,71]. Third, B-arrestin induces beta receptor internalization. The internalized receptors are transported to lysosomes for degradation (Figure 3) [72,73,74,75]. 

The second mechanism of catecholamine-induced autonomic dysfunction is a decline in baroreceptor sensitivity. Based on the hypothesis that heart rate and blood pressure variability may reflect the functionality of the baroreceptors, many studies have evaluated the effect of catecholamine exposure on baroreceptor functionality. In an animal study, autonomic control of blood vessels and the heart were found to be impaired during late sepsis compared to early sepsis. This autonomic dysfunction might be due to impairment in baroreceptors. [76]. In a clinical study by Pontet et al., heart rate variability was found to be significantly reduced in septic shock patients with multiple-organ dysfunction syndrome compared to those without multiple organ dysfunction syndrome. Reduction in heart rate variability can be a useful marker in identifying septic patients with high risk of developing multiple-organ dysfunction [77]. This can be explained by impaired baroreflex sensitivity that leads to decreased variability in heart rate. This demonstrates that the impaired functionality of baroreceptors is associated with prolonged catecholamine exposure. 

#### 2.1.4. Short-Acting Beta-Blockers as a Promising Medication in Patients with Septic Shock 

In an animal study by Suzuki et al., Esmolol improved myocardial oxygen utilization and helped preserve myocardial function in rats with peritonitis-induced septic shock. Rats with Esmolol infusion were found to have less lactic acid elevation, less tumor necrosis factor-alpha concentration, and better cardiac output [78]. This pre-clinical study demonstrates the potential protective role of Esmolol via increasing cardiac efficiency and oxygen utilization. Another animal study discussed the effect of Esmolol on renal blood flow in septic sheep. They found that Esmolol reduced renal blood flow by reducing renal perfusion pressure [79]. This raises a concern about using Esmolol in critically ill patients, especially those with renal dysfunction. More clinical research is needed to assess Esmolol’s effect on renal function. 

In a meta-analysis of randomized clinical trials in 2022, Zhang et al. found that Esmolol may significantly decrease 28-day mortality in patients with septic shock. Esmolol was also found to be effective in controlling heart rate and having a cardioprotective role. No adverse effects on tissue perfusion, lung injury, or oxygen utilization were observed [80]. This highlights that Esmolol is effective, safe, and protective in patients with septic shock. This may be explained by the beta-blocker effect against catecholamine-induced autonomic and immunologic dysfunction discussed earlier. 

In 2023, the Italian Society of Anesthesia, Analgesia, Resuscitation, and Intensive Care (SIAARTI)’s experts stated that beta-blockers have protective effects in critically ill patients with increased heart rate and adrenergic effect to protect against organ dysfunction. They recommended ruling out possible causes of tachycardia first before starting beta-blockers [81]. However, the 2021 guidelines did not discuss the use of beta-blockers or other autonomic dysfunction modulators in septic patients [19]. Beta-blocker use should be discussed in future revisions of sepsis guidelines. 

Another short-acting beta-blocker that has been studied is Landilol. Landilol is a very short-acting beta-blocker that is eight times more selective to beta-1 receptors than esmolol. In a randomized controlled trial in Japan, Landilol was used in patients with septic shock who were treated with catecholamines and developed atrial flutter, atrial fibrillation, or sinus tachycardia [82]. They found that Landilol was effective in treating patients with tachyarrhythmias and reducing the risk of developing new episodes of tachyarrhythmia in those patients. Despite assessing the safety and efficacy of Landilol in this clinical trial, the effect on organ damage or overall morality was not assessed [82]. In the STRESS-L clinical trial in 2023, Whitehouse et al. used Landilol in adults with septic shock and tachycardia who were treated with norepinephrine for more than 24 hours. They found that Landilol did not improve organ function, measured by the sequential organ failure assessment score (SOFA score), over 24 days after randomization [83].

#### 2.1.5. Long-Acting Beta-Blockers as a Promising Medication in Patients with Septic Shock 

In a recent study in 2023, Ge et al. found that starting long-acting beta-blockers in patients with septic shock was associated with reduced 28-day and 90-day mortality, while using a short-acting beta-blocker (Esmolol) in those patients was not associated with reduced mortality [84]. However, the results of this study are not consistent with the results of the previous meta-analysis by Zhang et al. [80]. This highlights the importance of conducting more large-size clinical studies to assess the significance of Esmolol in septic patients.

#### 2.1.6. Continuing Chronic Beta-Blockers in Patients with Acute Septic Shock 

If a patient is a long-term beta-blocker user, is it beneficial to continue the beta-blocker after admitting the patient to the ICU for septic shock? Interestingly, continuing beta-blockers was found to be beneficial for patients with or without septic shock after ICU admission, as will be discussed later. 

Patients with chronic beta-blocker use were found to have a lower rate of mortality after developing sepsis, compared to patients not taking beta-blockers prior to admission. Christensen et al. found that patients with preadmission chronic use of beta-blockers have a better 30-day mortality following ICU admission compared to beta-blocker non-users [85]. In a systematic review in 2019 by Tan et al., chronic beta-blocker exposure before developing sepsis was found to be associated with less mortality in septic patients [86]. Kuo et al. classified chronic beta-blocker users based on the selectivity of their beta-blockers and they found that chronic use of selective beta-1 is associated with improved mortality in patients with septic shock. In contrast, users of non-selective beta-blockers did not have significant improvement in mortality [87]. This finding supports the protective role of selective beta-blockers in patients with septic shock. These studies highlight the protective effect of chronic pre-admission beta-blockers on patients’ mortality. This was compelling enough to evaluate the effect of continuing beta-blockers after admission. Notably, Fuchs et al. found that continuing chronic beta-blockers is associated with improved 90-day mortality in septic patients, compared to patients with beta-blocker cessation [88].

### 2.2. Effect of Beta-Blockers in Patients with Electrical Storm 

An electrical storm is defined as three or more episodes of ventricular fibrillation (VF), pulseless ventricular tachycardia (pVT), or shocks from an implantable cardioverter defibrillation (ICD) separated by at least 5 minutes within 24 hours [89,90]. Electrical storms can happen in up to 40% of patients with ICDs. Developing an electrical storm is more common in patients with chronic renal failure, low ejection fraction (EF), and prior myocardial infarction. Patients who develop electrical storm have a higher mortality than those with isolated episodes of pVT or VF [90,91,92,93,94,95,96]. Electrical storms are thought to occur secondary to structural heart disease, channelopathies, and re-entry due to scar formation [96]. Those patients with electrical storm have a shockable rhythm. However, a subset of those patients do not respond to regular Advanced Cardiovascular Life Support (ACLS) management, including defibrillation, epinephrine, and antiarrhythmic medications [97]. As a result, there is a new trend in using promising therapies for refractory pVT and VF. These therapies include beta-blockers, double sequential defibrillation, deep sedation, extracorporeal cardiopulmonary resuscitation, and stellate ganglion blockade [94,98,99,100,101,102,103,104,105]. 

Current European Society of Cardiology class I recommendations for patients with electrical storm include using mild to moderate sedation and a non-selective beta-blocker plus amiodarone. Isoproterenol or transvenous pacing is recommended for patients with QT prolongation and recurrent torsades des pointes despite correcting potential causes and giving magnesium. Catheter ablation is also recommended for electrical storm caused by a monomorphic ventricular tachycardia (MMVT) refractory to anti-arrhythmic medications. Class IIa recommendations for electrical storm that is refractory to medications include deep sedation and intubation. Catheter ablation is also recommended for recurrent polymorphic ventricular tachycardia (PMVT) and VF that are unresponsive to medications or coronary revascularization. Class IIb recommendations include using Quinidine in recurrent PMVT who fail other medications, autonomic modulation, and mechanical circulatory support [96,106]. Autonomic modulation can be achieved by giving beta-blockers or performing stellate ganglion blockade in patients who do not respond to beta-blockers [97,105,106,107,108]. Herein, we highlight the role of beta-blockers as autonomic modulation agents to treat patients with an electrical storm. 

Theoretically, adrenergic stimulation may have stimulating effects in triggering electrical storm; subsequently, giving antiadrenergic drugs, such as beta-blockers, is reasonable to break this cycle of frequent adrenergic stimuli. Herein, we highlight the promising role of beta-blockers in treating patients with electrical storms. In a systematic review of experimental animal studies in 2012 discussing the potential role of beta-blockers in pVT/VF, beta-blockers were found to be promising therapies in terminating pVT/VF, improving post-resuscitation myocardial function, decreasing arrhythmia frequency, and improving survival. This effect is thought to be because of the ability of beta-blockers to reduce oxygen requirements and the number of shocks required for return of spontaneous circulation (ROSC) [109]. The first systematic review and meta-analysis of clinical studies in 2019 included three clinical studies discussing beta-blocker effect on electrical storm management [94,97,98,99,110]. Gottlieb et al. found that beta-blockers were associated with improved mortality and morbidity, such as survival-to-discharge, survival with a favorable neurologic outcome, survival-to-admission, and ROSC [97].

#### 2.2.1. Effect of Short-Acting Beta-Blockers in Patients with Electrical Storm

In a recent animal study in 2021, the effect of administering a single dose of Esmolol with epinephrine during cardiac resuscitation of induced VT in pigs was assessed. Ruggeri et al. found that coronary perfusion pressure was higher in the Esmolol group compared to the control group. They also found that pigs treated with Esmolol had less cortical degeneration and necrosis [111]. This demonstrates the potential protective role of Esmolol during resuscitation. 

Driver et al. used Esmolol in patients with refractory pVT and VF who received at least three shocks, 3 mg of epinephrine, and 300 mg of amiodarone. Patients treated with Esmolol were found to have a higher incidence of ROSC compared to those who did not receive Esmolol. The Esmolol group was also found to have higher rates of sustained ROSC, survival to discharge with a favorable neurologic outcome, survival to hospital discharge, and survival to intensive care unit admission [94]. In another clinical study by Lee et al., patients with at least three defibrillation shocks, 3 mg of epinephrine, and 300 mg of amiodarone were given Esmolol. ROSC incidence was higher in the Esmolol group compared to the control group [99]. These two clinical studies are observational retrospective studies; however, there is no randomized clinical trial comparing the usage of Esmolol versus control. Large clinical trials are encouraged to evaluate the effectiveness of Esmolol in refractory VT and VF. 

According to the class IIb recommendations of the European Society of Cardiology, autonomic modulation using intravenous beta-blockers, such as Esmolol, is recommended in patients with refractory electrical storm as a late step in resuscitation. The journal of the American College of Cardiology’s recent review recommends using intravenous beta-blockers, such as Esmolol, later in resuscitation as the second step out of a three-step guideline [96,106]. Furthermore, many reported cases showed an effective role of intravenous Esmolol in regaining ROSC when it was used earlier in electrical storm resuscitation [112,113]. In a recent study by Lian et al., Esmolol was given to patients with refractory pVT and VF early and directly after ACLS procedures. They found that the duration from cardiac arrest to Esmolol injection was shorter in the survival group compared to the deceased group [114]. This highlights the importance of giving Esmolol early in patients with electrical storm after applying ACLS procedures.

Landilol is another short-acting beta-blocker that was evaluated as a potential treatment of electrical storm. In a clinical trial by Miwa et al., Landilol was used to treat patients with electrical storm refractory to defibrillation, epinephrine, and amiodarone. Landilol was found to be effective in terminating electrical storm in 80% of patients who were resistant to epinephrine and amiodarone [115]. More clinical trials are encouraged to evaluate the effect of Landilol and compare it to Esmolol.

#### 2.2.2. Effect of Long-Acting Beta-Blockers in Patients with Electrical Storm

The European Society of Cardiology class I recommendations suggest adding a non-selective beta-blocker to amiodarone in treating patients with refractory electrical storm [106]. Chatzidou et al. compared between adding a nonselective beta-blocker (propranolol) versus a beta-1 selective blocker (metoprolol) to amiodarone in treating patients with ICD who have developed an electrical storm. They found that patients treated with propranolol have a lower rate of ventricular arrhythmias compared to patients treated with metoprolol. They also found that propranolol is more effective in terminating electrical storm [116]. This study suggests that amiodarone plus propranolol is more effective in terminating electrical storm in patients with ICD compared to the combination of amiodarone and metoprolol.

### 2.3. Beta-Blockers in Acute Heart Failure (AHF)

Beta-blockers are known to be the main therapy in the management of patients with heart failure with reduced ejection fraction (HFrEF) [117,118,119]. After developing left ventricular dysfunction, activation of both the renin–angiotensin–aldosterone system (RAS), and the SNS happens as a response to heart failure-induced hypoperfusion. Sustained stimulation of the sympathetic nervous system and high levels of catecholamines result in beta receptor desensitization and downregulation. As a result, this may lead to reduced systolic function, increased risk of developing ventricular arrhythmia, and an accelerated remodeling process. Giving beta-blockers to those patients protects against the adverse consequences of sustained sympathetic stimulation and the continuous activation of beta receptors. Subsequently, beta-blockers help in beta receptor upregulation, increasing the cardiac inotropic reserve, and suppressing the cardiotoxic and modeling effects of RAS and SNS [9,57,120,121,122,123,124,125]. 

Beta-blockers are considered the first-line treatment in patients with HFrEF according to current guidelines. However, there is an underuse of beta-blockers because of many misconceptions. These misconceptions include the false idea that beta-blockers should only be used in chronic compensated HFrEF and in patients who do not have specific co-morbidities that are thought to be absolute contraindications to beta-blockers. There is also a misconception that all beta-blockers will lead to the deterioration of the hemodynamic status in patients with decompensated heart failure because of their effect in reducing heart rate and blood pressure [126,127,128,129,130,131]. Moreover, there are no current guidelines explaining when and how physicians should stop or continue beta-blockers in patients with ADHF or cardiogenic shock who are already on chronic beta-blockers. Additionally, there are no guidelines discussing starting beta-blockers in decompensated patients treated with inotropes [118,128].

#### 2.3.1. Beta-Blocker Effect on Hemodynamic Status and Cardiac Index

A false belief about beta-blockers is that all beta-blockers have a negative effect on cardiac index and hemodynamic status. This leads to the underuse of beta-blockers in patients with HFrEF. Beta-blockers can be classified into three categories. Each type of beta-blocker has a different effect on hemodynamic status, cardiac index, and blood pressure. First-generation beta-blockers, such as propranolol, are non-selective beta-blockers that affect both beta-1 and beta-2 receptors equally. This type of beta-blocker can cause a negative ionotropic effect through blocking beta-1 receptors and increase systemic vascular resistance (SVR) via the blockage of beta-2 receptors. As a result, they may lead to a significant decrease in cardiac output. None of the beta-blockers in this group is recommended in HFrEF [128,129,130].

Second-generation beta-blockers, such as bisoprolol, metoprolol, and nebivolol, are selective beta-blockers that mainly affect beta-1 receptors. They are preferred in patients with chronic obstructive pulmonary disease or mild asthma. Additionally, nebivolol is preferred in patients with arterial hypertension because it facilitates nitric oxide release. Second-generation beta-blockers have a lesser effect on the cardiac index than first-generation beta-blockers. Both first and second generations have no significant effect on pulmonary capillary wedge pressure (PCWP). 

Third-generation beta-blockers, such as carvedilol and bucindolol, can be selective or non-selective with vasodilatory effects. This generation has the ability to reduce SVR, potentially increase the cardiac index, and reduce PCWP. Beta-blockers can also be classified into lipophilic and hydrophilic agents. All beta-blockers approved for heart failure are lipophilic and do not require dosage adjustment in patients with reduced renal function [128,129,130].

#### 2.3.2. Continuing Chronic Beta-Blockers in Patients with Acute Decompensated Heart Failure (ADHF)

In a systematic review and meta-analysis discussing the potential outcomes of discontinuing chronic beta-blockers in patients with acute decompensated heart failure (ADHF), one clinical trial and five retrospective studies were included [131]. In the randomized clinical trial, Jondeau et al. compared continuing beta-blockers versus discontinuing them in patients with an EF of below 40% who were previously using beta-blockers and developed ADHF. They found that continuing beta-blockers is not associated with acute deterioration or delay in improvement [129]. Beta-blocker discontinuation in four observational studies was found to be associated with increased risk of mortality and rehospitalization in patients with decompensated heart failure [132,133,134]. In another study by Orso et al., they found that continuing beta-blockers in patients with chronic beta-blocker use before admission and starting beta-blockers in patients who did not take beta-blockers before admission were both associated with a decreased rate of in-hospital mortality [135]. The meta-analysis of these studies shows that continuing beta-blockers in ADHF is associated with decreased mortality and rehospitalization. It recommends continuing beta-blockers in admitted patients with ADHF if it is clinically appropriate [131]. Further clinical trials should be conducted to evaluate the effect of discontinuing chronic beta-blockers in patients with ADHF and define the clinical characteristics that warrant discontinuing beta-blockers in patients with ADHF.

#### 2.3.3. Continuing Chronic Beta-Blockers in ADHF Patients Treated with Inotropes

Gattis et al. found that discontinuing beta-blockers in ADHF patients treated with milrinone is associated with an increased risk of mortality and rehospitalization [134]. Bohm et al. studied the effects of continuing beta-blockers in patients hospitalized with ADHF who have received inotropes. Bohm et al. found that continuing beta-blockers in ADHF patients treated with dobutamine or levosimendan showed a decreased risk of death up to 180 days. However, this reduction in risk of death is not statistically significant [136]. More clinical trials are necessary to evaluate the effect of continuing or discontinuing beta-blockers in patients with ADHF who required inotropes during hospitalization. 

### 2.4. Beta-Blockers in Cardiogenic Shock (CS)

CS is defined as developing inadequate tissue perfusion as a result of decreased cardiac output [137]. Severe ADHF is considered one of the causes of CS. In general, CS is classified based on etiology into two categories, including acute myocardial infarction (AMI)-induced CS as well as non-AMI-induced CS that may result from other etiologies, such as cardiomyopathies, myocarditis, arrhythmias, ADHF, myocardial depression from septic or neurogenic shock, myocardial stunning following cardiac arrest, or congenital defects [138,139,140].

Treatment of CS differs according to etiology. However, the mutual feature in CS is the presence of myocardial dysfunction. Theoretically, treating CS can be achieved by optimizing preload, decreasing afterload, and achieving the optimal contractility of the affected ventricle. There are two goals that should be considered while treating CS. The first goal is achieving adequate tissue perfusion, and the second goal is minimizing cardiac work. There should be a balance between these two goals. 

Inotropes are medicines that increase myocardial contractility. They are classified based on their mechanism of action into the following: (1) calcium sensitizers, such as Levosimendan, (2) phosphodiesterase III inhibitors, such as Milrinone, and (3) adrenergic agonists, such as norepinephrine, epinephrine, dobutamine, and dopamine. Inotropes can be also classified into inodilators causing vasodilation, such as Milrinone and dobutamine, and inopressors causing vasoconstriction, such as norepinephrine, epinephrine, and Levosimendan [141,142]. 

In ADHF-induced CS, an inodilator, ideally dobutamine, is preferred along with a potential vasopressor, ideally norepinephrine, to compensate the vasodilation effect of dobutamine. As we mentioned earlier, the effect of epinephrine alone is equal to the effect of norepinephrine and dobutamine combined. However, using epinephrine is associated with worse outcomes and increased mortality. As a result, dobutamine plus norepinephrine is preferred over epinephrine alone. In contrast to ADHF-induced CS, vasopressors, ideally norepinephrine, are preferred over inotropes in AMI-induced CS as inotropes may cause worsening cardiac ischemia [35,37,118,141,142,143,144,145]. 

Despite the different management of CS due to the diversity of its etiology, using beta-blockers seems to be unwanted due to their undesirable hemodynamic effects. However, many recent studies report a protective role of beta-blockers in patients with CS. Di Santo et al. evaluated the hemodynamic effects of receiving beta-blockers in the 24 hours before developing CS. They found that beta-blockers were not associated with impaired hemodynamic status in cardiac care unit patients with CS who required inotropic therapy, including dobutamine and milrinone [146]. In another study by Cardelli et al., they found that there was no association between using beta-blockers and increased rate of all-cause mortality in patients with CS due to different causes. Additionally, they found that discontinuing beta-blockers is associated with increased mortality up to one year [127]. This demonstrates the promising role of beta-blockers in patients with CS. In contrast to the previous studies, Ryu et al. found that concurrent usage of beta-blockers with inotropes or vasopressors is not associated with decreased mortality rates in patients with CS [147]. Large randomized clinical trials are needed to evaluate the role of starting or continuing beta-blockers in patients with CS and its effect on mortality and morbidity.

### 2.5. Beta-Blockers in Severe Traumatic Brain Injury (TBI)

TBI includes multiple pathologies to the brain that can result from primary insult or subsequent secondary injury due to increased intracranial pressure (ICP). A new trend in TBI management is finding potential therapies that can decrease the secondary insult by minimizing the undesired effects of high ICP. Increased ICP due to secondary injury may result in decreased cerebral blood perfusion, ischemia, and brain herniation [148,149]. Catecholamine surge after TBI is a stimulus of secondary injury, leading to increased severity of brain injury [150,151]. Theoretically, suppressing this catecholamine or sympathetic surge may decrease the severity of the secondary insult and improve mortality rates in patients with TBI.

Many clinical studies have shown a significant improvement in outcomes and mortality in patients with TBI who were treated with beta-blockers [147,152,153,154,155,156,157,158,159]. Three observational studies showed improved functional outcome measured by Glasgow Outcome Score-Extended (GOS-E) at acute care discharge, while two studies showed improved GOS-E at ≥ 6 months [155,160,161]. Moreover, Asmar et al. found that beta-blockers decrease posttraumatic hyperthermia by reducing the frequency of febrile episodes, decreasing the maximum rise in temperature in patients with TBI. They also found that propranolol is superior to other the beta-blockers they used in reducing the maximum temperature and the number of febrile episodes [162].

In a randomized clinical trial by Schroeppel et al., Glasgow Coma Scale (GCS) at the end of the study was significantly improved in TBI patients treated with propranolol [157]. In another randomized clinical trial, TBI patients who were treated with propranolol were found to have lower mortality than patients who were not treated with propranolol. They also suggested routine administration of oral propranolol in neuro-intensive care units for patients with TBI [160]. In a study by Ley et al., propranolol was found to be superior to other beta-blockers in decreasing mortality in TBI patients [161]. In another study by Schroeppel et al., patients treated with propranolol had a lower rate of mortality compared to patients treated with other beta-blockers, although the propranolol group had lower GCS scores at the time of admission [163]. A recent systematic review and meta-analysis in 2023 by Hart et al. showed that using beta-blockers was associated with lower in-hospital mortality rates and better functional outcomes in patients with TBI [164]. Therefore, we encourage the practice of using propranolol in patients with TBI if clinically appropriate.

## 3. Conclusions

Sympathetic overstimulation may lead to autonomic dysfunction, adrenergic receptor insensitivity, and deterioration of hemodynamic status. It may also lead to immunologic dysfunction. All these adverse consequences are undesired in critically ill patients, such as those with septic shock, cardiogenic shock, and ADHF. Sympathetic overstimulation may also be the stimulus of ventricular arrhythmias and electrical storms. Suppressing adrenergic overstimulation may be a method to break this electrical storm. Sympathetic overstimulation may also lead to an increase in the severity of secondary injury in TBI.

Beta-blockers have been shown to be a promising therapy in decreasing mortality in many critically ill patients. Their anti-adrenergic effect compensates against the sympathetic surge in many pathologies, including septic shock, electrical storm, cardiogenic shock, heart failure, and TBI. We encourage clinicians to consider using beta-blockers in treating these pathologies when clinically appropriate. More clinical trials are encouraged to evaluate the outcome of using beta-blockers in critically ill patients and define the best way to find a balance between the benefits of sympathetic stimulation and disadvantages of sympathetic overstimulation.

## References

[1] Gibbons C.H. (2019). Basics of Autonomic Nervous System Function. Handbook of Clinical Neurology.

[2] Gatica S., Aravena D., Echeverría C., Santibanez J.F., Riedel C.A., Simon F., Simon F., Bernabeu C. (2023). Effects of Adrenergic Receptor Stimulation on Human Hemostasis: A Systematic Review. Advances in Molecular Pathology.

[3] Shields R.W. (1993). Functional Anatomy of the Autonomic Nervous System. J. Clin. Neurophysiol..

[4] Karemaker J.M. (2017). An Introduction into Autonomic Nervous Function. Physiol. Meas..

[5] Wehrwein E.A., Orer H.S., Barman S.M., Terjung R. (2016). Overview of the Anatomy, Physiology, and Pharmacology of the Autonomic Nervous System. Comprehensive Physiology.

[6] Benarroch E.E. (2020). Physiology and Pathophysiology of the Autonomic Nervous System. Contin. Lifelong Learn. Neurol..

[7] Johnson M. (1998). The β-Adrenoceptor. Am. J. Respir. Crit. Care Med..

[8] Cotecchia S. (2010). The α_1_-Adrenergic Receptors: Diversity of Signaling Networks and Regulation. J. Recept. Signal Transduct..

[9] Bencivenga L., Liccardo D., Napolitano C., Visaggi L., Rengo G., Leosco D. (2019). β-Adrenergic Receptor Signaling and Heart Failure. Heart Fail. Clin..

[10] McGraw D.W. (2005). Molecular Mechanisms of 2-Adrenergic Receptor Function and Regulation. Proc. Am. Thorac. Soc..

[11] Reid J.L. (1986). Alpha-Adrenergic Receptors and Blood Pressure Control. Am. J. Cardiol..

[12] Liu X., Luo D., Zhang J., Du L. (2020). Distribution and Relative Expression of Vasoactive Receptors on Arteries. Sci. Rep..

[13] Walch L., Brink C., Norel X. (2001). The Muscarinic Receptor Subtypes in Human Blood Vessels. Therapie.

[14] Kanagy N.L. (2005). A2-Adrenergic Receptor Signalling in Hypertension. Clin. Sci..

[15] Carrara M., Ferrario M., Bollen Pinto B., Herpain A. (2021). The Autonomic Nervous System in Septic Shock and Its Role as a Future Therapeutic Target: A Narrative Review. Ann. Intensive Care.

[16] Stolk R.F., Van Der Pasch E., Naumann F., Schouwstra J., Bressers S., Van Herwaarden A.E., Gerretsen J., Schambergen R., Ruth M.M., Van Der Hoeven J.G. (2020). Norepinephrine Dysregulates the Immune Response and Compromises Host Defense during Sepsis. Am. J. Respir. Crit. Care Med..

[17] Ferreira J.A., Bissell B.D. (2018). Misdirected Sympathy: The Role of Sympatholysis in Sepsis and Septic Shock. J. Intensive Care Med..

[18] Dellinger R.P., Carlet J.M., Masur H., Gerlach H., Calandra T., Cohen J., Gea-Banacloche J., Keh D., Marshall J.C., Parker M.M. (2004). Surviving Sepsis Campaign Guidelines for Management of Severe Sepsis and Septic Shock. Crit. Care Med..

[19] Evans L., Rhodes A., Alhazzani W., Antonelli M., Coopersmith C.M., French C., Machado F.R., Mcintyre L., Ostermann M., Prescott H.C. (2021). Surviving Sepsis Campaign: International Guidelines for Management of Sepsis and Septic Shock 2021. Intensive Care Med..

[20] Rhodes A., Evans L.E., Alhazzani W., Levy M.M., Antonelli M., Ferrer R., Kumar A., Sevransky J.E., Sprung C.L., Nunnally M.E. (2017). Surviving Sepsis Campaign: International Guidelines for Management of Sepsis and Septic Shock: 2016. Crit. Care Med..

[21] Dellinger R.P., Levy M.M., Carlet J.M., Bion J., Parker M.M., Jaeschke R., Reinhart K., Angus D.C., Brun-Buisson C., Beale R. (2008). Surviving Sepsis Campaign: International Guidelines for Management of Severe Sepsis and Septic Shock: 2008. Crit. Care Med..

[22] Srzić I., Adam V.N., Pejak D.T. (2022). Sepsis Definition: What’s New in the Treatment Guidelines. Acta Clin. Croat..

[23] Somers V.K., Mark A.L., Abboud F.M. (1991). Interaction of Baroreceptor and Chemoreceptor Reflex Control of Sympathetic Nerve Activity in Normal Humans. J. Clin. Investig..

[24] Chapleau M.W., Li Z., Meyrelles S.S., Ma X., Abboud F.M. (2001). Mechanisms Determining Sensitivity of Baroreceptor Afferents in Health and Disease. Ann. N. Y. Acad. Sci..

[25] Desai T.H., Collins J.C., Snell M., Mosqueda-Garcia R. (1997). Modeling of Arterial and Cardiopulmonary Baroreflex Control of Heart Rate. Am. J. Physiol.-Heart Circ. Physiol..

[26] Halliwill J.R., Morgan B.J., Charkoudian N. (2003). Peripheral Chemoreflex and Baroreflex Interactions in Cardiovascular Regulation in Humans. J. Physiol..

[27] O’regan R.G., Majcherczyk S. (1982). Role of Peripheral Chemoreceptors and Central Chemosensitivity in the Regulation of Respiration and Circulation. J. Exp. Biol..

[28] Shi R., Hamzaoui O., De Vita N., Monnet X., Teboul J.-L. (2020). Vasopressors in Septic Shock: Which, When, and How Much?. Ann. Transl. Med..

[29] Senatore F., Jagadeesh G., Rose M., Pillai V.C., Hariharan S., Liu Q., Tzu-Yun M., Sapru M.K., Southworth M.R., Stockbridge N. (2019). FDA Approval of Angiotensin II for the Treatment of Hypotension in Adults with Distributive Shock. Am. J. Cardiovasc. Drugs.

[30] Ruslan M., Baharuddin K., Noor N., Yazid M., Md Noh A.Y., Rahman A. (2021). Norepinephrine in Septic Shock: A Systematic Review and Meta-Analysis. West. J. Emerg. Med..

[31] Russell J.A., Walley K.R., Singer J., Gordon A.C., Hébert P.C., Cooper D.J., Holmes C.L., Mehta S., Granton J.T., Storms M.M. (2008). Vasopressin versus Norepinephrine Infusion in Patients with Septic Shock. N. Engl. J. Med..

[32] Sedhai Y.R., Shrestha D.B., Budhathoki P., Memon W., Acharya R., Gaire S., Pokharel N., Maharjan S., Jasaraj R., Sodhi A. (2022). Vasopressin versus Norepinephrine as the First-Line Vasopressor in Septic Shock: A Systematic Review and Meta-Analysis. J. Clin. Transl. Res..

[33] Liu Z.-M., Chen J., Kou Q., Lin Q., Huang X., Tang Z., Kang Y., Li K., Zhou L., Study Group of Investigators (2018). Terlipressin versus Norepinephrine as Infusion in Patients with Septic Shock: A Multicentre, Randomised, Double-Blinded Trial. Intensive Care Med..

[34] Laterre P.-F., Berry S.M., Blemings A., Carlsen J.E., François B., Graves T., Jacobsen K., Lewis R.J., Opal S.M., Perner A. (2019). Effect of Selepressin vs Placebo on Ventilator- and Vasopressor-Free Days in Patients With Septic Shock: The SEPSIS-ACT Randomized Clinical Trial. JAMA.

[35] Bougouin W., Slimani K., Renaudier M., Binois Y., Paul M., Dumas F., Lamhaut L., Loeb T., Ortuno S., Deye N. (2022). Epinephrine versus Norepinephrine in Cardiac Arrest Patients with Post-Resuscitation Shock. Intensive Care Med..

[36] Dribin T.E., Waserman S., Turner P.J. (2023). Who Needs Epinephrine? Anaphylaxis, Autoinjectors, and Parachutes. J. Allergy Clin. Immunol. Pract..

[37] Belletti A., Nagy A., Sartorelli M., Mucchetti M., Putzu A., Sartini C., Morselli F., De Domenico P., Zangrillo A., Landoni G. (2020). Effect of Continuous Epinephrine Infusion on Survival in Critically Ill Patients: A Meta-Analysis of Randomized Trials. Crit. Care Med..

[38] Motiejunaite J., Deniau B., Blet A., Gayat E., Mebazaa A. (2022). Inotropes and Vasopressors Are Associated with Increased Short-Term Mortality but Not Long-Term Survival in Critically Ill Patients. Anaesth. Crit. Care Pain Med..

[39] Belletti A., Castro M.L., Silvetti S., Greco T., Biondi-Zoccai G., Pasin L., Zangrillo A., Landoni G. (2015). The Effect of Inotropes and Vasopressors on Mortality: A Meta-Analysis of Randomized Clinical Trials. Br. J. Anaesth..

[40] Ostrowski S.R., Gaïni S., Pedersen C., Johansson P.I. (2015). Sympathoadrenal Activation and Endothelial Damage in Patients with Varying Degrees of Acute Infectious Disease: An Observational Study. J. Crit. Care.

[41] Takenaka M.C., Guereschi M.G., Basso A.S. (2017). Neuroimmune Interactions: Dendritic Cell Modulation by the Sympathetic Nervous System. Semin. Immunopathol..

[42] Steinman L. (2004). Elaborate Interactions between the Immune and Nervous Systems. Nat. Immunol..

[43] Kenney M.J., Ganta C.K., Terjung R. (2014). Autonomic Nervous System and Immune System Interactions. Comprehensive Physiology.

[44] Kizaki T., Shirato K., Sakurai T., Ogasawara J., Oh-ishi S., Matsuoka T., Izawa T., Imaizumi K., Haga S., Ohno H. (2009). Β2-Adrenergic Receptor Regulate Toll-like Receptor 4-Induced Late-Phase NF-κB Activation. Mol. Immunol..

[45] Spengler R.N., Chensue S.W., Giacherio D.A., Blenk N., Kunkel S.L. (1994). Endogenous Norepinephrine Regulates Tumor Necrosis Factor-Alpha Production from Macrophages in Vitro. J. Immunol..

[46] Van Der Poll T., Jansen J., Endert E., Sauerwein H.P., Van Deventer S.J. (1994). Noradrenaline Inhibits Lipopolysaccharide-Induced Tumor Necrosis Factor and Interleukin 6 Production in Human Whole Blood. Infect. Immun..

[47] Van Der Poll T., Coyle S.M., Barbosa K., Braxton C.C., Lowry S.F. (1996). Epinephrine Inhibits Tumor Necrosis Factor-Alpha and Potentiates Interleukin 10 Production during Human Endotoxemia. J. Clin. Investig..

[48] Ağaç D., Estrada L.D., Maples R., Hooper L.V., Farrar J.D. (2018). The Β2-Adrenergic Receptor Controls Inflammation by Driving Rapid IL-10 Secretion. Brain Behav. Immun..

[49] Verhoeckx K.C.M., Doornbos R.P., Van Der Greef J., Witkamp R.F., Rodenburg R.J.T. (2005). Inhibitory Effects of the *β*_2_-adrenergic Receptor Agonist Zilpaterol on the LPS-induced Production of TNF-*α* in Vitro and in Vivo. J. Vet. Pharmacol. Ther..

[50] Izeboud C.A., Monshouwer M., Van Miert A.S.J.P.A.M., Witkamp R.F. (1999). The β-Adrenoceptor Agonist Clenbuterol Is a Potent Inhibitor of the LPS-Induced Production of TNF-α and IL-6 in Vitro and in Vivo. Inflamm. Res..

[51] Lemaire L.C., De Kruif M.D., Giebelen I.A., Levi M., Van Der Poll T., Heesen M. (2006). Dobutamine Does Not Influence Inflammatory Pathways during Human Endotoxemia. Crit. Care Med..

[52] Kohm A.P., Sanders V.M. (2001). Norepinephrine and Beta 2-Adrenergic Receptor Stimulation Regulate CD4+ T and B Lymphocyte Function in Vitro and in Vivo. Pharmacol. Rev..

[53] Dessauer C.W. (2009). Adenylyl Cyclase–A-Kinase Anchoring Protein Complexes: The Next Dimension in cAMP Signaling. Mol. Pharmacol..

[54] Vandamme J., Castermans D., Thevelein J.M. (2012). Molecular Mechanisms of Feedback Inhibition of Protein Kinase A on Intracellular cAMP Accumulation. Cell. Signal..

[55] Dünser M.W., Hasibeder W.R. (2009). Sympathetic Overstimulation During Critical Illness: Adverse Effects of Adrenergic Stress. J. Intensive Care Med..

[56] Bucher M., Kees F., Taeger K., Kurtz A. (2003). Cytokines Down-Regulate A1-Adrenergic Receptor Expression during Endotoxemia. Crit. Care Med..

[57] Bernardin G., Strosberg A.D., Bernard A., Mattei M., Marullo S. (1998). β-Adrenergic Receptor-Dependent and -Independent Stimulation of Adenylate Cyclase Is Impaired during Severe Sepsis in Humans. Intensive Care Med..

[58] Cariou A., Pinsky M.R., Monchi M., Laurent I., Vinsonneau C., Chiche J.-D., Charpentier J., Dhainaut J.-F. (2008). Is Myocardial Adrenergic Responsiveness Depressed in Human Septic Shock?. Intensive Care Med..

[59] Rudiger A., Singer M. (2016). Decatecholaminisation during Sepsis. Crit. Care.

[60] Wu L.-L., Yang S.-L., Yang R.-C., Hsu H.-K., Hsu C., Dong L.-W., Liu M.-S. (2003). G Protein and Adenylate Cyclase Complex-Mediated Signal Transduction in the Rat Heart During Sepsis. Shock.

[61] Jiang H., Galtes D., Wang J., Rockman H.A. (2022). G Protein-Coupled Receptor Signaling: Transducers and Effectors. Am. J. Physiol.-Cell Physiol..

[62] Marzano F., Rapacciuolo A., Ferrara N., Rengo G., Koch W.J., Cannavo A. (2021). Targeting GRK5 for Treating Chronic Degenerative Diseases. Int. J. Mol. Sci..

[63] Claing A. (2002). Endocytosis of G Protein-Coupled Receptors: Roles of G Protein-Coupled Receptor Kinases and ß-Arrestin Proteins. Prog. Neurobiol..

[64] Port J.D. (2023). Dissecting Beta-Adrenergic Receptors. JACC Basic Transl. Sci..

[65] Fan X., Gu X., Zhao R., Zheng Q., Li L., Yang W., Ding L., Xue F., Fan J., Gong Y. (2016). Cardiac Β2-Adrenergic Receptor Phosphorylation at Ser355/356 Regulates Receptor Internalization and Functional Resensitization. PLoS ONE.

[66] Skalhegg B.S. (2000). Specificity in the cAMP/PKA Signaling Pathway. Differential Expression, Regulation, and Subcellular Localization of Subunits of PKA. Front. Biosci..

[67] Jenei-Lanzl Z., Zwingenberg J., Lowin T., Anders S., Straub R.H. (2015). Proinflammatory Receptor Switch from Gαs to Gαi Signaling by β-Arrestin-Mediated PDE4 Recruitment in Mixed RA Synovial Cells. Brain Behav. Immun..

[68] Shenoy S.K., Drake M.T., Nelson C.D., Houtz D.A., Xiao K., Madabushi S., Reiter E., Premont R.T., Lichtarge O., Lefkowitz R.J. (2006). β-Arrestin-Dependent, G Protein-Independent ERK1/2 Activation by the Β2 Adrenergic Receptor. J. Biol. Chem..

[69] Giembycz M.A. (1996). Phosphodiesterase 4 and Tolerance to Beta 2-Adrenoceptor Agonists in Asthma. Trends Pharmacol. Sci..

[70] Essayan D.M. (2001). Cyclic Nucleotide Phosphodiesterases. J. Allergy Clin. Immunol..

[71] Lorton D., Bellinger D. (2015). Molecular Mechanisms Underlying β-Adrenergic Receptor-Mediated Cross-Talk between Sympathetic Neurons and Immune Cells. Int. J. Mol. Sci..

[72] Tian X., Kang D.S., Benovic J.L., Gurevich V.V. (2014). β-Arrestins and G Protein-Coupled Receptor Trafficking. Arrestins—Pharmacology and Therapeutic Potential.

[73] Laporte S.A., Oakley R.H., Zhang J., Holt J.A., Ferguson S.S., Caron M.G., Barak L.S. (1999). The Beta2-Adrenergic Receptor/Betaarrestin Complex Recruits the Clathrin Adaptor AP-2 during Endocytosis. Proc. Natl. Acad. Sci. USA.

[74] Reiter E., Lefkowitz R.J. (2006). GRKs and β-Arrestins: Roles in Receptor Silencing, Trafficking and Signaling. Trends Endocrinol. Metab..

[75] Ménard L., Ferguson S.S.G., Barak L.S., Bertrand L., Premont R.T., Colapietro A.-M., Lefkowitz R.J., Caron M.G. (1996). Members of the G Protein-Coupled Receptor Kinase Family That Phosphorylate the β_2_-Adrenergic Receptor Facilitate Sequestration. Biochemistry.

[76] Pancoto J.A.T., Corrêa P.B.F., Oliveira-Pelegrin G.R., Rocha M.J.A. (2008). Autonomic Dysfunction in Experimental Sepsis Induced by Cecal Ligation and Puncture. Auton. Neurosci..

[77] Pontet J., Contreras P., Curbelo A., Medina J., Noveri S., Bentancourt S., Migliaro E.R. (2003). Heart Rate Variability as Early Marker of Multiple Organ Dysfunction Syndrome in Septic Patients. J. Crit. Care.

[78] Suzuki T., Morisaki H., Serita R., Yamamoto M., Kotake Y., Ishizaka A., Takeda J. (2005). Infusion of the β-Adrenergic Blocker Esmolol Attenuates Myocardial Dysfunction in Septic Rats. Crit. Care Med..

[79] Loon L.M., Rongen G.A., Hoeven J.G., Veltink P.H., Lemson J. (2019). β-Blockade Attenuates Renal Blood Flow in Experimental Endotoxic Shock by Reducing Perfusion Pressure. Physiol. Rep..

[80] Zhang J., Chen C., Liu Y., Yang Y., Yang X., Yang J. (2022). Benefits of Esmolol in Adults with Sepsis and Septic Shock: An Updated Meta-Analysis of Randomized Controlled Trials. Medicine.

[81] Guarracino F., Cortegiani A., Antonelli M., Behr A., Biancofiore G., Del Gaudio A., Forfori F., Galdieri N., Grasselli G., Paternoster G. (2023). The Role of Beta-Blocker Drugs in Critically Ill Patients: A SIAARTI Expert Consensus Statement. J. Anesth. Analg. Crit. Care.

[82] Kakihana Y., Nishida O., Taniguchi T., Okajima M., Morimatsu H., Ogura H., Yamada Y., Nagano T., Morishima E., Matsuda N. (2020). Efficacy and Safety of Landiolol, an Ultra-Short-Acting Β1-Selective Antagonist, for Treatment of Sepsis-Related Tachyarrhythmia (J-Land 3S): A Multicentre, Open-Label, Randomised Controlled Trial. Lancet Respir. Med..

[83] Whitehouse T., Hossain A., Perkins G.D., Gordon A.C., Bion J., Young D., McAuley D., Singer M., Lord J., Gates S. (2023). Landiolol and Organ Failure in Patients With Septic Shock: The STRESS-L Randomized Clinical Trial. JAMA.

[84] Ge C.-L., Zhang L.-N., Ai Y.-H., Chen W., Ye Z.-W., Zou Y., Peng Q.-Y. (2023). Effect of β-Blockers on Mortality in Patients with Sepsis: A Propensity-Score Matched Analysis. Front. Cell. Infect. Microbiol..

[85] Christensen S., Johansen M., Tønnesen E., Larsson A., Pedersen L., Lemeshow S., Sørensen H. (2011). Preadmission Beta-Blocker Use and 30-Day Mortality among Patients in Intensive Care: A Cohort Study. Crit. Care.

[86] Tan K., Harazim M., Tang B., Mclean A., Nalos M. (2019). The Association between Premorbid Beta Blocker Exposure and Mortality in Sepsis—A Systematic Review. Crit. Care.

[87] Kuo M.-J., Chou R.-H., Lu Y.-W., Guo J.-Y., Tsai Y.-L., Wu C.-H., Huang P.-H., Lin S.-J. (2021). Premorbid Β1-Selective (but Not Non-Selective) β-Blocker Exposure Reduces Intensive Care Unit Mortality among Septic Patients. J. Intensive Care.

[88] Fuchs C., Wauschkuhn S., Scheer C., Vollmer M., Meissner K., Kuhn S.-O., Hahnenkamp K., Morelli A., Gründling M., Rehberg S. (2017). Continuing Chronic Beta-Blockade in the Acute Phase of Severe Sepsis and Septic Shock Is Associated with Decreased Mortality Rates up to 90 Days. Br. J. Anaesth..

[89] Al-Khatib S.M., Stevenson W.G., Ackerman M.J., Bryant W.J., Callans D.J., Curtis A.B., Deal B.J., Dickfeld T., Field M.E., Fonarow G.C. (2018). 2017 AHA/ACC/HRS Guideline for Management of Patients With Ventricular Arrhythmias and the Prevention of Sudden Cardiac Death. J. Am. Coll. Cardiol..

[90] Dyer S., Mogni B., Gottlieb M. (2020). Electrical Storm: A Focused Review for the Emergency Physician. Am. J. Emerg. Med..

[91] Credner S.C., Klingenheben T., Mauss O., Sticherling C., Hohnloser S.H. (1998). Electrical Storm in Patients with Transvenous Implantable Cardioverter-Defibrillators. J. Am. Coll. Cardiol..

[92] Arya A., Haghjoo M., Dehghani M.R., Fazelifar A.F., Nikoo M.-H., Bagherzadeh A., Sadr-Ameli M.A. (2006). Prevalence and Predictors of Electrical Storm in Patients With Implantable Cardioverter-Defibrillator. Am. J. Cardiol..

[93] Huang D.T., Traub D. (2008). Recurrent Ventricular Arrhythmia Storms in the Age of Implantable Cardioverter Defibrillator Therapy: A Comprehensive Review. Prog. Cardiovasc. Dis..

[94] Driver B.E., Debaty G., Plummer D.W., Smith S.W. (2014). Use of Esmolol after Failure of Standard Cardiopulmonary Resuscitation to Treat Patients with Refractory Ventricular Fibrillation. Resuscitation.

[95] Bänsch D., Böcker D., Brunn J., Weber M., Breithardt G., Block M. (2000). Clusters of Ventricular Tachycardias Signify Impaired Survival in Patients with Idiopathic Dilated Cardiomyopathy and Implantable Cardioverter Defibrillators. J. Am. Coll. Cardiol..

[96] Jentzer J.C., Noseworthy P.A., Kashou A.H., May A.M., Chrispin J., Kabra R., Arps K., Blumer V., Tisdale J.E., Solomon M.A. (2023). Multidisciplinary Critical Care Management of Electrical Storm: JACC State-of-the-Art Review. J. Am. Coll. Cardiol..

[97] Gottlieb M., Dyer S., Peksa G.D. (2020). Beta-Blockade for the Treatment of Cardiac Arrest Due to Ventricular Fibrillation or Pulseless Ventricular Tachycardia: A Systematic Review and Meta-Analysis. Resuscitation.

[98] Nademanee K., Taylor R., Bailey W.E., Rieders D.E., Kosar E.M. (2000). Treating Electrical Storm: Sympathetic Blockade Versus Advanced Cardiac Life Support–Guided Therapy. Circulation.

[99] Lee Y.H., Lee K.J., Min Y.H., Ahn H.C., Sohn Y.D., Lee W.W., Oh Y.T., Cho G.C., Seo J.Y., Shin D.H. (2016). Refractory Ventricular Fibrillation Treated with Esmolol. Resuscitation.

[100] Cheskes S., Verbeek P.R., Drennan I.R., McLeod S.L., Turner L., Pinto R., Feldman M., Davis M., Vaillancourt C., Morrison L.J. (2022). Defibrillation Strategies for Refractory Ventricular Fibrillation. N. Engl. J. Med..

[101] Deakin C.D., Morley P., Soar J., Drennan I.R. (2020). Double (Dual) Sequential Defibrillation for Refractory Ventricular Fibrillation Cardiac Arrest: A Systematic Review. Resuscitation.

[102] Martins R.P., Urien J.-M., Barbarot N., Rieul G., Sellal J.-M., Borella L., Clementy N., Bisson A., Guenancia C., Sagnard A. (2020). Effectiveness of Deep Sedation for Patients With Intractable Electrical Storm Refractory to Antiarrhythmic Drugs. Circulation.

[103] Ubben J.F.H., Heuts S., Delnoij T.S.R., Suverein M.M., Hermanides R.C., Otterspoor L.C., Kraemer C.V.E., Vlaar A.P.J., Van Der Heijden J.J., Scholten E. (2024). Favorable Resuscitation Characteristics in Patients Undergoing Extracorporeal Cardiopulmonary Resuscitation: A Secondary Analysis of the INCEPTION-Trial. Resusc. Plus.

[104] Yukawa T., Kashiura M., Sugiyama K., Tanabe T., Hamabe Y. (2017). Neurological Outcomes and Duration from Cardiac Arrest to the Initiation of Extracorporeal Membrane Oxygenation in Patients with Out-of-Hospital Cardiac Arrest: A Retrospective Study. Scand. J. Trauma Resusc. Emerg. Med..

[105] Tian Y., Wittwer E.D., Kapa S., McLeod C.J., Xiao P., Noseworthy P.A., Mulpuru S.K., Deshmukh A.J., Lee H.-C., Ackerman M.J. (2019). Effective Use of Percutaneous Stellate Ganglion Blockade in Patients With Electrical Storm. Circ. Arrhythm. Electrophysiol..

[106] Zeppenfeld K., Tfelt-Hansen J., De Riva M., Winkel B.G., Behr E.R., Blom N.A., Charron P., Corrado D., Dagres N., De Chillou C. (2022). 2022 ESC Guidelines for the Management of Patients with Ventricular Arrhythmias and the Prevention of Sudden Cardiac Death. Eur. Heart J..

[107] Malik V., Shivkumar K. (2024). Stellate Ganglion Blockade for the Management of Ventricular Arrhythmia Storm. Eur. Heart J..

[108] Takahashi K., Egami Y., Nishino M., Tanouchi J. (2024). Clinical Impact of Stellate Ganglion Phototherapy on Ventricular Tachycardia Storm Requiring Mechanical Circulatory Support Devices: A Case Report. Eur. Heart J.—Case Rep..

[109] De Oliveira F.C., Feitosa-Filho G.S., Ritt L.E.F. (2012). Use of Beta-Blockers for the Treatment of Cardiac Arrest Due to Ventricular Fibrillation/Pulseless Ventricular Tachycardia: A Systematic Review. Resuscitation.

[110] Long D.A., Long B., April M.D. (2020). Does β-Blockade for Treatment of Refractory Ventricular Fibrillation or Pulseless Ventricular Tachycardia Improve Outcomes?. Ann. Emerg. Med..

[111] Ruggeri L., Nespoli F., Ristagno G., Fumagalli F., Boccardo A., Olivari D., Affatato R., Novelli D., De Giorgio D., Romanelli P. (2021). Esmolol during Cardiopulmonary Resuscitation Reduces Neurological Injury in a Porcine Model of Cardiac Arrest. Sci. Rep..

[112] Liu B., Xie B., Chen X., Zhu K., Wang C.-M., Guo S.-H. (2022). A Successful Case of Electrical Storm Rescue after Acute Myocardial Infarction. BMC Cardiovasc. Disord..

[113] Agrawal A., Cardinale M., Frenia D., Dalia T., Shah C. (2020). Esmolol Use in Dual Axis Defibrillation Resistant Ventricular Fibrillation. Case Rep. Cardiol..

[114] Lian R., Zhang G., Yan S., Sun L., Gao W., Yang J., Li G., Huang R., Wang X., Liu R. (2022). The First Case Series Analysis on Efficacy of Esmolol Injection for In-Hospital Cardiac Arrest Patients with Refractory Shockable Rhythms in China. Front. Pharmacol..

[115] Miwa Y., Ikeda T., Mera H., Miyakoshi M., Hoshida K., Yanagisawa R., Ishiguro H., Tsukada T., Abe A., Yusu S. (2010). Effects of Landiolol, an Ultra-Short-Acting .BETA.1-Selective Blocker, on Electrical Storm Refractory to Class III Antiarrhythmic Drugs. Circ. J..

[116] Chatzidou S., Kontogiannis C., Tsilimigras D.I., Georgiopoulos G., Kosmopoulos M., Papadopoulou E., Vasilopoulos G., Rokas S. (2018). Propranolol Versus Metoprolol for Treatment of Electrical Storm in Patients With Implantable Cardioverter-Defibrillator. J. Am. Coll. Cardiol..

[117] Johri N., Matreja P.S., Maurya A., Varshney S. (2023). Smritigandha Role of β-Blockers in Preventing Heart Failure and Major AdverseCardiac Events Post Myocardial Infarction. Curr. Cardiol. Rev..

[118] McDonagh T.A., Metra M., Adamo M., Gardner R.S., Baumbach A., Böhm M., Burri H., Butler J., Čelutkienė J., Authors/Task Force Members (2022). 2021 ESC Guidelines for the Diagnosis and Treatment of Acute and Chronic Heart Failure: Developed by the Task Force for the Diagnosis and Treatment of Acute and Chronic Heart Failure of the European Society of Cardiology (ESC). With the Special Contribution of the Heart Failure Association (HFA) of the ESC. Eur. J. Heart Fail..

[119] Maddox T.M., Januzzi J.L., Allen L.A., Breathett K., Butler J., Davis L.L., Fonarow G.C., Ibrahim N.E., Lindenfeld J., Masoudi F.A. (2021). 2021 Update to the 2017 ACC Expert Consensus Decision Pathway for Optimization of Heart Failure Treatment: Answers to 10 Pivotal Issues About Heart Failure With Reduced Ejection Fraction. J. Am. Coll. Cardiol..

[120] Mann D.L., Bristow M.R. (2005). Mechanisms and Models in Heart Failure: The Biomechanical Model and Beyond. Circulation.

[121] Mudd J.O., Kass D.A. (2008). Tackling Heart Failure in the Twenty-First Century. Nature.

[122] Triposkiadis F., Karayannis G., Giamouzis G., Skoularigis J., Louridas G., Butler J. (2009). The Sympathetic Nervous System in Heart Failure. J. Am. Coll. Cardiol..

[123] Armour J.A. (2004). Cardiac Neuronal Hierarchy in Health and Disease. Am. J. Physiol.-Regul. Integr. Comp. Physiol..

[124] Liaudet L., Calderari B., Pacher P. (2014). Pathophysiological Mechanisms of Catecholamine and Cocaine-Mediated Cardiotoxicity. Heart Fail. Rev..

[125] Brouri F., Findji L., Mediani O., Mougenot N., Hanoun N., Le Naour G., Hamon M., Lechat P. (2002). Toxic Cardiac Effects of Catecholamines: Role of β-Adrenoceptor Downregulation. Eur. J. Pharmacol..

[126] Bangalore S., Makani H., Radford M., Thakur K., Toklu B., Katz S.D., DiNicolantonio J.J., Devereaux P.J., Alexander K.P., Wetterslev J. (2014). Clinical Outcomes with β-Blockers for Myocardial Infarction: A Meta-Analysis of Randomized Trials. Am. J. Med..

[127] Cardelli L.S., Cherbi M., Huet F., Schurtz G., Bonnefoy-Cudraz E., Gerbaud E., Bonello L., Leurent G., Puymirat E., Casella G. (2023). Beta Blockers Improve Prognosis When Used Early in Patients with Cardiogenic Shock: An Analysis of the FRENSHOCK Multicenter Prospective Registry. Pharmaceuticals.

[128] Masarone D., Martucci M.L., Errigo V., Pacileo G. (2021). The Use of β-Blockers in Heart Failure with Reduced Ejection Fraction. J. Cardiovasc. Dev. Dis..

[129] Jondeau G., Milleron O. (2015). Beta-Blockers in Acute Heart Failure. JACC Heart Fail..

[130] Schurtz G., Mewton N., Lemesle G., Delmas C., Levy B., Puymirat E., Aissaoui N., Bauer F., Gerbaud E., Henry P. (2023). Beta-Blocker Management in Patients Admitted for Acute Heart Failure and Reduced Ejection Fraction: A Review and Expert Consensus Opinion. Front. Cardiovasc. Med..

[131] Prins K.W., Neill J.M., Tyler J.O., Eckman P.M., Duval S. (2015). Effects of Beta-Blocker Withdrawal in Acute Decompensated Heart Failure. JACC Heart Fail..

[132] Butler J., Young J.B., Abraham W.T., Bourge R.C., Adams K.F., Clare R., O’Connor C. (2006). Beta-Blocker Use and Outcomes Among Hospitalized Heart Failure Patients. J. Am. Coll. Cardiol..

[133] Fonarow G.C., Abraham W.T., Albert N.M., Stough W.G., Gheorghiade M., Greenberg B.H., O’Connor C.M., Sun J.L., Yancy C.W., Young J.B. (2008). Influence of Beta-Blocker Continuation or Withdrawal on Outcomes in Patients Hospitalized With Heart Failure. J. Am. Coll. Cardiol..

[134] Gattis W.A., O’Connor C.M., Leimberger J.D., Felker G.M., Adams K.F., Gheorghiade M. (2003). Clinical Outcomes in Patients on Beta-Blocker Therapy Admitted with Worsening Chronic Heart Failure. Am. J. Cardiol..

[135] Orso F., Baldasseroni S., Fabbri G., Gonzini L., Lucci D., D’Ambrosi C., Gobbi M., Lecchi G., Randazzo S., Masotti G. (2009). Role of Beta-blockers in Patients Admitted for Worsening Heart Failure in a Real World Setting: Data from the Italian Survey on Acute Heart Failure. Eur. J. Heart Fail..

[136] Böhm M., Link A., Cai D., Nieminen M.S., Filippatos G.S., Salem R., Solal A.C., Huang B., Padley R.J., Kivikko M. (2011). Beneficial Association of β-Blocker Therapy on Recovery from Severe Acute Heart Failure Treatment: Data from the Survival of Patients With Acute Heart Failure in Need of Intravenous Inotropic Support Trial. Crit. Care Med..

[137] Saunders S.L., Clifford L.M., Meere W. (2024). Cardiogenic Shock without Hypotension in Acute Severe Primary Mitral Regurgitation: A Case Report. Oxf. Med. Case Rep..

[138] Chien S.-C., Wang C.-A., Liu H.-Y., Lin C.-F., Huang C.-Y., Chien L.-N. (2024). Comparison of the Prognosis among In-Hospital Survivors of Cardiogenic Shock Based on Etiology: AMI and Non-AMI. Ann. Intensive Care.

[139] Chien S.-C., Hsu C.-Y., Liu H.-Y., Lin C.-F., Hung C.-L., Huang C.-Y., Chien L.-N. (2021). Cardiogenic Shock in Taiwan from 2003 to 2017 (CSiT-15 Study). Crit. Care.

[140] Shah M., Patnaik S., Patel B., Ram P., Garg L., Agarwal M., Agrawal S., Arora S., Patel N., Wald J. (2018). Trends in Mechanical Circulatory Support Use and Hospital Mortality among Patients with Acute Myocardial Infarction and Non-Infarction Related Cardiogenic Shock in the United States. Clin. Res. Cardiol..

[141] Polyzogopoulou E., Arfaras-Melainis A., Bistola V., Parissis J. (2020). Inotropic Agents in Cardiogenic Shock. Curr. Opin. Crit. Care.

[142] Bistola V., Arfaras-Melainis A., Polyzogopoulou E., Ikonomidis I., Parissis J. (2019). Inotropes in Acute Heart Failure: From Guidelines to Practical Use: Therapeutic Options and Clinical Practice. Card. Fail. Rev..

[143] Shankar A., Gurumurthy G., Sridharan L., Gupta D., Nicholson W.J., Jaber W.A., Vallabhajosyula S. (2022). A Clinical Update on Vasoactive Medication in the Management of Cardiogenic Shock. Clin. Med. Insights Cardiol..

[144] Tarvasmäki T., Lassus J., Varpula M., Sionis A., Sund R., Køber L., Spinar J., Parissis J., Banaszewski M., The CardShock Study Investigators (2016). Current Real-Life Use of Vasopressors and Inotropes in Cardiogenic Shock—Adrenaline Use Is Associated with Excess Organ Injury and Mortality. Crit. Care.

[145] Ibánez B., James S., Agewall S., Antunes M.J., Bucciarelli-Ducci C., Bueno H., Caforio A.L.P., Crea F., Goudevenos J.A., Halvorsen S. (2017). 2017 ESC Guidelines for the Management of Acute Myocardial Infarction in Patients Presenting with ST-Segment Elevation. Rev. Esp. Cardiol. Engl. Ed..

[146] Di Santo P., Mathew R., Jung R.G., Simard T., Skanes S., Mao B., Ramirez F.D., Marbach J.A., Abdel-Razek O., Motazedian P. (2021). Impact of Baseline Beta-Blocker Use on Inotrope Response and Clinical Outcomes in Cardiogenic Shock: A Subgroup Analysis of the DOREMI Trial. Crit. Care.

[147] Ryu R., Hauschild C., Bahjri K., Tran H. (2022). The Usage of Concomitant Beta-Blockers with Vasopressors and Inotropes in Cardiogenic Shock. Med. Sci..

[148] Eraky A.M., Treffy R., Hedayat H.S. (2023). Cisternostomy as a Surgical Treatment for Traumatic Brain Injury-Related Prolonged and Delayed Intracranial Pressure Elevation: A Case Report. Cureus.

[149] Eraky A.M., Treffy R., Hedayat H.S. (2022). Cisternotomy and Liliequist’s Membrane Fenestration as a Surgical Treatment for Idiopathic Intracranial Hypertension (Pseudotumor Cerebri): A Case Report. Cureus.

[150] Naredi S., Lambert G., Edén E., Zäll S., Runnerstam M., Rydenhag B., Friberg P. (2000). Increased Sympathetic Nervous Activity in Patients With Nontraumatic Subarachnoid Hemorrhage. Stroke.

[151] Borlongan C., Acosta S., De La Pena I., Tajiri N., Kaneko Y., Lozano D., Gonzales-Portillo G. (2015). Neuroinflammatory Responses to Traumatic Brain Injury: Etiology, Clinical Consequences, And&nbsp;Therapeutic Opportunities. Neuropsychiatr. Dis. Treat..

[152] Cotton B.A., Snodgrass K.B., Fleming S.B., Carpenter R.O., Kemp C.D., Arbogast P.G., Morris J.A. (2007). Beta-Blocker Exposure Is Associated With Improved Survival After Severe Traumatic Brain Injury. J. Trauma Inj. Infect. Crit. Care.

[153] Inaba K., Teixeira P.G.R., David J.-S., Chan L.S., Salim A., Brown C., Browder T., Beale E., Rhee P., Demetriades D. (2008). Beta-Blockers in Isolated Blunt Head Injury. J. Am. Coll. Surg..

[154] Edavettal M., Gross B.W., Rittenhouse K., Alzate J., Rogers A., Estrella L., Miller J.A., Rogers F.B. (2016). An Analysis of Beta-Blocker Administration Pre-and Post-Traumatic Brain Injury with Subanalyses for Head Injury Severity and Myocardial Injury. Am. Surg..

[155] Ahl R., Thelin E.P., Sjölin G., Bellander B.-M., Riddez L., Talving P., Mohseni S. (2017). β-Blocker after Severe Traumatic Brain Injury Is Associated with Better Long-Term Functional Outcome: A Matched Case Control Study. Eur. J. Trauma Emerg. Surg..

[156] Mohseni S., Talving P., Thelin E.P., Wallin G., Ljungqvist O., Riddez L. (2015). The Effect of Β-blockade on Survival After Isolated Severe Traumatic Brain Injury. World J. Surg..

[157] Schroeppel T.J., Sharpe J.P., Shahan C.P., Clement L.P., Magnotti L.J., Lee M., Muhlbauer M., Weinberg J.A., Tolley E.A., Croce M.A. (2019). Beta-Adrenergic Blockade for Attenuation of Catecholamine Surge after Traumatic Brain Injury: A Randomized Pilot Trial. Trauma Surg. Acute Care Open.

[158] Zangbar B., Khalil M., Rhee P., Joseph B., Kulvatunyou N., Tang A., Friese R.S., O’Keeffe T. (2016). Metoprolol Improves Survival in Severe Traumatic Brain Injury Independent of Heart Rate Control. J. Surg. Res..

[159] Ko A., Harada M.Y., Barmparas G., Thomsen G.M., Alban R.F., Bloom M.B., Chung R., Melo N., Margulies D.R., Ley E.J. (2016). Early Propranolol after Traumatic Brain Injury Is Associated with Lower Mortality. J. Trauma Acute Care Surg..

[160] Khalili H., Ahl R., Paydar S., Sjolin G., Cao Y., Abdolrahimzadeh Fard H., Niakan A., Hanna K., Joseph B., Mohseni S. (2020). Beta-Blocker Therapy in Severe Traumatic Brain Injury: A Prospective Randomized Controlled Trial. World J. Surg..

[161] Ley E.J., Leonard S.D., Barmparas G., Dhillon N.K., Inaba K., Salim A., O’Bosky K.R., Tatum D., Azmi H., Ball C.G. (2018). Beta Blockers in Critically Ill Patients with Traumatic Brain Injury: Results from a Multicenter, Prospective, Observational American Association for the Surgery of Trauma Study. J. Trauma Acute Care Surg..

[162] Asmar S., Bible L., Chehab M., Tang A., Khurrum M., Castanon L., Ditillo M., Douglas M., Joseph B. (2021). Traumatic Brain Injury Induced Temperature Dysregulation: What Is the Role of β Blockers?. J. Trauma Acute Care Surg..

[163] Schroeppel T.J., Sharpe J.P., Magnotti L.J., Weinberg J.A., Clement L.P., Croce M.A., Fabian T.C. (2014). Traumatic Brain Injury and β-Blockers: Not All Drugs Are Created Equal. J. Trauma Acute Care Surg..

[164] Hart S., Lannon M., Chen A., Martyniuk A., Sharma S., Engels P.T. (2023). Beta Blockers in Traumatic Brain Injury: A Systematic Review and Meta-Analysis. Trauma Surg. Acute Care Open.

